# Arsenic Concentrations and Dietary Exposure in Rice-Based Infant Food in Australia

**DOI:** 10.3390/ijerph17020415

**Published:** 2020-01-08

**Authors:** Zhuyun Gu, Shamali de Silva, Suzie M. Reichman

**Affiliations:** 1 School of Engineering, RMIT University, Melbourne 3001, Australia; s3460282@student.rmit.edu.au (Z.G.); shamali.desilva@rmit.edu.au (S.d.S.); 2 Centre for Environmental Sustainability and Remediation, RMIT University, Melbourne 3001, Australia

**Keywords:** arsenic, dietary intake, dietary exposure, inorganic arsenic, *Oryza sativa*, food safety, rice-based food, baby food

## Abstract

Rice-based products are widely used to feed infants and young children. However, the association of rice-based products and high arsenic (As) concentrations have been investigated in a number of studies, but there is limited information from Australia. Therefore, the purpose of this study was to determine the As concentration and dietary exposure in infant rice milk, cereal, crackers and pasta as well as to investigate the relationship between As concentration and rice content, rice type and product origin. Total arsenic (tAs) concentrations were determined by nitric acid digestion and ICP-MS while inorganic arsenic (iAs) was determined by acid extraction, followed by ICP-MS with an interfaced hydride generation system. Nearly 75% of samples had inorganic As exceeding the EU maximum levels for infants and children (0.1 mg kg^−1^) and the mean iAs percentage of total reached as high as 84.8%. High tAs concentration was positively correlated with rice content and also related to brown (wholegrain). Estimates of dietary exposure showed that infants consuming large amounts of rice pasta or crackers will have an increased risk of health impact associated with excess intake of As through dietary exposure. Moreover, the current Australian guidelines for As in rice (1 mg kg^−1^) are above the WHO or EU guideline and therefore, will be less protective of high sensitivity consumers like infants and children.

## 1. Introduction

Arsenic (As), a naturally occurring metalloid, is widely distributed in air, soil and groundwater in both organic and inorganic forms. Current knowledge indicates that organic forms of As have relatively low toxicity, but inorganic As (iAs) is a non-threshold human carcinogen [1]. In South-East Asia (e.g., Bangladesh, West Bengal and Vietnam) where groundwater is a major source of As contamination; it has adversely affected tens of millions of people [2]. In addition to exposure through As-contaminated drinking groundwater, people can also be exposed to As through food ingestion, particularly rice [3,4].

Rice (*Oryza sativa*) plants accumulate As more than similar cereal crops [5]. It has been suggested that rice’s high As absorption is due to a high-affinity phosphate/arsenate uptake system [6,7]. Additionally, the anaerobic paddy soil culture of rice plant also contributes to high As accumulation in rice because phosphate uptake in saturated soil environments does not have the diffusion limitations observed in drier soil [5]. The predominant As species found in rice are arsenate (As (V)), arsenite (As (III)) and dimethylarsinic acid (DMA) [8,9,10].

Rice and rice-based baby foods are frequently used to feed infants and young children due to the bland taste, iron fortification and relatively low allergic potential [11]. Thus, there is the potential for higher levels of As exposure in infants and young children than the general population. Infants and toddlers have a higher food consumption rate per body weight basis, which further exacerbates the risks of dietary exposure to As via rice for this age group [12].

Researchers have also reported that brown rice has higher concentrations of total arsenic (tAs) and iAs than white rice [9,13,14], with As being localized at the surface of brown rice compared with being dispersed throughout the grain in white rice [15]. In addition, organic rice has been shown to have higher iAs than inorganic rice, which could be associated with the inclusion of wholegrain rice in the organic product. This is of particular concern because baby food companies are increasingly shifting to organic products due to the association of organic food and being healthier and more nutritious [11].

Studies of As concentrations in infant rice-based products have reported elevated As exposure to infants and young children in many countries [9,11,16,17,18,19,20,21]. Despite the potential risk of As in infant rice-based food globally, there were no As guidelines developed specifically for this vulnerable age group until 2016, when the European Union set a maximum level iAs of 0.1 mg kg^−1^ for rice used in the production of food for infants and young children [22] (Table 1). In comparison, the current maximum level in Australia only includes tAs and was developed for adults. In addition, in Australia only one published study has investigated As in one baby rice-based product amongst general rice-based foods and found that >53% of tested products exceeded the European Union maximum level of 0.1 mg kg^−1^ [22,23].

Therefore, there is a need for further evaluation of As concentrations in infant foods that are available in Australia as well as to determine the potential infant exposure in order to ensure the protection of Australian children.

Hence, the objectives of this study were:

- to determine the tAs and iAs concentrations in rice-based infant foods for sale in Australia;
- to investigate how the characteristics of rice-based products (i.e., rice content, rice texture and origin) are related to arsenic concentrations;
- to calculate the dietary intake and exposure of infants to examine the potential As exposure risk to Australian infants.

## 2. Materials and Methods

### 2.1. Sample Collection

Thirty-nine samples representing four infant food categories: rice milk powder, rice cereal (including rice porridge and rice congee), rice crackers (including rice cakes and rice biscuits) and rice pasta were purchased from supermarkets in Melbourne Australia between April and May 2017. For rice milk powder and rice pasta, only one brand was found, thus, 6 samples of milk powder and wheat pasta with no rice content were purchased for comparison. Different supermarkets were selected so that three different batches of the same type of food were bought to maximise sample representativeness. Manufacture and expiry date of all samples were also recorded to ensure replicates of products were from different batches. The selection of products covered 11 brands from 6 countries of origin as well as various rice types, including brown, white, organic and inorganic rice. It was not possible to trace the origin(s) of samples labelled as “made in Australia from local and imported ingredients”. Therefore, they were separated as “Australia (mix)”.

### 2.2. Sample Preparation and Chemical Analysis

The food samples were dried in an oven at 60 °C for 48 h and homogenously ground using a ceramic mortar where necessary. Dry weight results were converted to fresh weight through moisture content. Moisture content was calculated by weight difference before and after drying divided by initial weight. Analytical results are expressed as fresh weight throughout this paper, unless specifically stated.

The experimental procedure for tAs testing was designed following the method of Fransisca et al. [29] with minor modifications. Briefly, 1.0 g dried and ground sample was weighed directly into a digestion tube and 6.0 mL 70% nitric acid (AR grade) was added. The samples were then heated at 100 °C for 2 h, then left to cool to room temperature before being diluted to 25 mL with ultrapure water (18 MΩ.cm), filtered through a 0.45 μm pore size cellulose acetate syringe filter (Membrane Solutions) and stored in polypropylene vials. A 1.25 mL aliquot of each sample was diluted with ultrapure water to a total volume of 10 mL before analysis by inductively coupled plasma mass spectrometer (ICP-MS, Agilent Technologies, California, USA, 7700× Analyser) for total As.

The majority of samples were further analysed for iAs after tAs analysis; however, rice cereal samples were not analysed for iAs due to low tAs concentration as consumed. The iAs analysis method was adapted from Holak and Specchio [30]. Briefly, 1 g of homogenised sample was weighed into a polypropylene tube and extracted with 7 mL 50% perchloric acid (AR grade) by heating on a hot block at 80 °C for 1 h. After extraction, 5 mL of sample extraction solution was transferred to a polypropylene tube and 4 mL 10 M hydrochloric acid (AR grade), 0.5 mL 48% hydrobromic acid (AR grade) and 0.5 mL 3% hydrazine sulphate were added. The solution was then determined by ICP-MS (Perkin Elmer Elan DRC II) with an interfaced hydride generation system.

### 2.3. Quality Control

For tAs determination, the instrumental limit of detection (LOD) for the ICP-MS was 0.02 µg kg^−1^, which was determined by 3 times the standard deviation of the counts in a blank solution. This gave a corresponding dry sample LOD of 0.004 mg kg^−1^. Four reagent blanks were included in each of the three batches of sample digestion and all 12 blanks were below LOD. Each food sample was analysed in duplicate. For 85% of samples, the relative percent difference (RPD) was within 10%. The rest of samples were within 25% except one sample (one non-rice pasta sample out of three replicates) which had a RPD of 42%. This high RPD was mostly likely due to higher errors as a result of the concentration being close to the LOD. The certified reference material (NCS ZC73031 Carrot) gave a recovery of 74% of the tAs certified concentrations (0.11 ± 0.02 mg kg^−1^).

For iAs determination, the sample LOD was 0.05 mg kg^−1^, which was determined through repeated analysis (approx. 10 measurements) of blank solutions and spiked solutions. One reagent blank was included in the iAs analysis and it was measured below the LOD. Samples were analysed in duplicate and the RPD was less than 5%. The recovery of reference material (AGAL40) and matrix spike were 105% and 97%, respectively. The iAs concentration of reference material (AGAL40) is 6.43 ± 0.22 mg kg^−1^.

### 2.4. Dietary Exposure Estimations

Dietary exposure estimates followed the method used by FAO/WHO [31]. Briefly, the principle equation for determining dietary intake was

DI = C × CR,(1)

DI = dietary intake (μg person^−1^ day^−1^),
C = concentration of contaminants (μg kg^−1^) and
CR = food consumption rate (kg person^−1^ day^−1^).

Dietary exposure was calculated using the following equation.

DE = DI/BW,(2)

DE = dietary exposure (μg kg^−1^ b. wt. day^−1^)
DI = dietary intake (μg person^−1^ day^−1^) and
BW = mean individual body weight (kg)

According to the 23rd Australia Total Diet Study (ATDS), mean rice and rice products consumption for 9 month-old infants was 8.6 g day^−1^ and for 2–5 year-old children, was 28 g day^−1^ [32]. Mean body weight was 8.9 kg for 9 month-old infants and 18 kg for 2–5 year-old children [32]. The upper 90th percentile for exposure was calculated at twice the mean exposure [31,32].

### 2.5. Reference Values for Comparison

The Australian current maximum level for tAs in rice (1.0 mg kg^−1^) is more than 3 times higher than the proposed WHO maximum levels for tAs (0.3 mg kg^−1^) [25]. Hence, we used the proposed WHO maximum levels as a tAs reference point in this study to compare to our results. However, it should be noted that the proposed maximum levels of 0.3 mg kg^−1^ for tAs was established for adults, which may not be protective for infants and young children. For iAs, the maximum iAs level (0.1 mg kg^−1^) that was specifically developed for infant rice products was used as an iAs reference point in our study [22].

For dietary exposure, there were no values relevant to infants and young children available worldwide; hence, we adopted the European benchmark dose lower confidence limit (BMDL) of 0.3 to 8 μg kg^−1^ b.w. per day [12]. It was developed by modelling the dose-response date from key epidemiological studies and using 1% extra risk as a benchmark response. Again, it should be noted that dietary exposure for infants and children can be 2–3 fold higher than that of adults [12] and thus, the BMDL may not be fully protective for infants and children.

### 2.6. Statistical Analysis

Statistical analyses were performed using Minitab^®^ Statistical Software (version 17, Minitab, LLC., Pennsylvania, USA) including descriptive statistics and box and whisker plots. Data were further evaluated via analysis of variance (ANOVA) and Fishers Least Significant Difference (LSD) to determine significant differences (*p* < 0.05) between treatment means. Regression lines were not included in figures where *p* > 0.05. Where As concentrations were <LOD, half of the sample LOD was used for statistical analysis [33].

## 3. Results and Discussion

### 3.1. As Occurrence in Australia Rice-Based Food

The tAs concentrations in infant rice-based food increased in the following order: rice milk powder^a^ > rice pasta^b^ > rice cereal^b^ > rice crackers^b^ (values with different superscript letters are significantly different, *p* < 0.05) (Table 2). Only tAs concentrations of rice milk powder exceeded the proposed maximum level of 0.3 mg kg^−1^ [25]. However, it is generally considered that rice milk powder would be diluted with water before consumption, which lowers the final tAs concentration. The comparison of infant rice products and infant non-rice-based products showed that the tAs concentration of rice foods was significantly higher than that of non-rice-based foods (*p* < 0.001), which strongly suggests that rice content contributes to As in infant foods. This finding was also observed by, for example, Signes-Pastor et al. [11] and Munera-Picazo et al. [19].

The iAs concentrations in rice-based infant foods were: rice milk powder^a^ > rice pasta^ab^ > rice crackers^b^ (values with different letters are significantly different, *P* < 0.05). Although ~88% of samples had tAs below the proposed maximum level of 0.3 mg kg^−1^ [25], nearly 75% of samples had iAs exceeding 0.1 mg kg^−1^ for infants and children [22]. This is of particular concern because iAs is considered more toxic than other As species. The mean iAs percentage reached as high as 84.8% of the tAs and the individual values ranged between 37% to 91%. Among the tested food groups, the iAs percentage of rice milk powder was significantly lower than that of rice pasta (*p* = 0.03). The significant variation in iAs percentage is potentially due to a number of factors, including the type of rice, soil arsenic speciation, soil organic matter and soil phosphorus content [6,34]. Our results are consistent with other studies globally (Table 3).

### 3.2. The Relationship between As Concentrations and Rice Properties and Other Factors

#### 3.2.1. The Relationship between tAs Concentration and Rice Content

**Figure 1:**
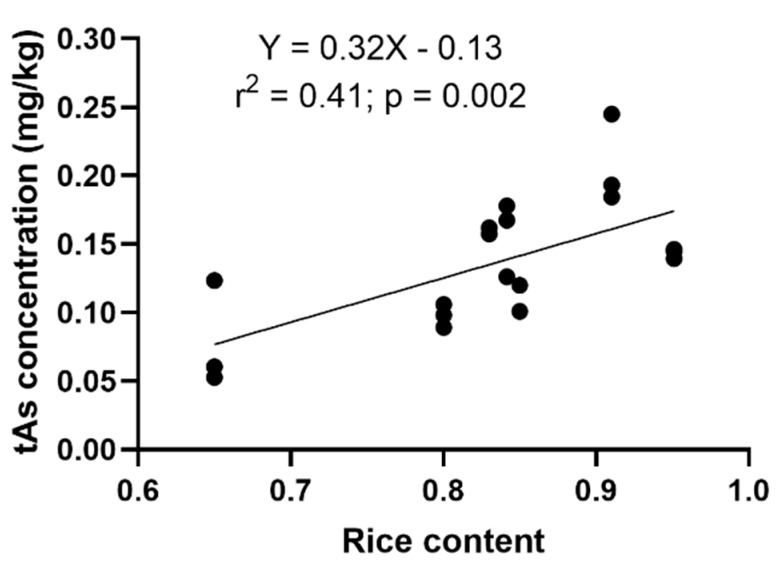
Relationship between total arsenic (tAs) concentration and the proportion of rice in infant rice crackers.

Rice crackers showed a significant positive relationship between the tAs concentrations and the proportion of rice in the crackers (r^2^ = 0.41; *p* = 0.002), which strongly suggests that rice is an important contributor to the As concentration of rice crackers (Figure 1). A similar positive relationship between As in infant rice food and the proportion of rice has been previously reported by Signes-Pastor et al. [11] and Munera-Picazo et al. [19].

Other food groups (rice milk; rice pasta and rice cereal) had poor correlations (r^2^ < 0.10; *p* > 0.05) between tAs and the proportion of rice in the food (Figure S1). We believe this is likely to be due to the low variance in rice content (mostly around 99%) in these food groups rather than that a relationship does not exist.

#### 3.2.2. The Relationship between iAs and tAs Concentrations

**Figure 2:**
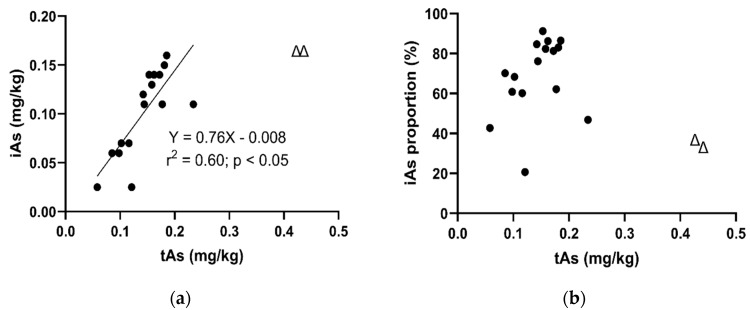
Relationships between (**a**) inorganic arsenic (iAs) and total arsenic (tAs) concentrations and (**b**) iAs as a proportion of tAs and tAs concentration for rice-based infant foods in Australia. Symbol Δ represents sample outlier and was not included in the linear regression analysis.

When tAs concentration was between 0.05 and 0.2 mg kg^−1^, iAs increased linearly with tAs (Figure 2a, r^2^ = 0.60; *p* = 0.0004). Such positive correlations between tAs and iAs concentrations in infant foods have been observed in previous studies [9,18,19]. Interestingly, in our study, there were two apparent outliers (represent as symbol Δ) with tAs around 0.4 mg kg^−1^ and iAs of 0.16 mg kg^−1^ that were not included in the regression (Figure 2a). Meharg et al. [17] reported a similar finding where iAs in baby rice increased linearly with tAs and then plateaued at the level of 0.16 mg kg^−1^. It seems that there is a maximum concentration of iAs in rice-based food; however, other studies have found iAs concentrations greater than 0.16 mg kg^−1^. One possible explanation is that because the international maximum level of iAs in rice (for adults) is 0.2 mg kg^−1^ [26], it is likely that rice product manufacturers will ensure the iAs concentration in their products is at or just below 0.2 mg kg^−1^. Thus, the plateau may be an artifact of the maximum level being 0.2 mg kg^−1^ rather than being related to rice physiology. However, further work is required to establish this.

Although the proportion of iAs against tAs did not show a significant linear relationship (Figure 2b, r^2^ = 0.09; *p* > 0.05), there is a general increasing trend when tAs concentration is between 0 and 0.2 mg kg^−1^. There were two apparent outliers (represent as symbol Δ), which were the same two outliers in Figure 2a and were not included in the regression line. This plot showed that iAs percentages are much more varied among different types of food. This variation could be as a result of a number of environmental variables, including the type of rice, soil arsenic speciation, soil organic matter or soil phosphorus content [6,34].

#### 3.2.3. The Relationship between tAs Concentration and Rice Type

**Figure 3:**
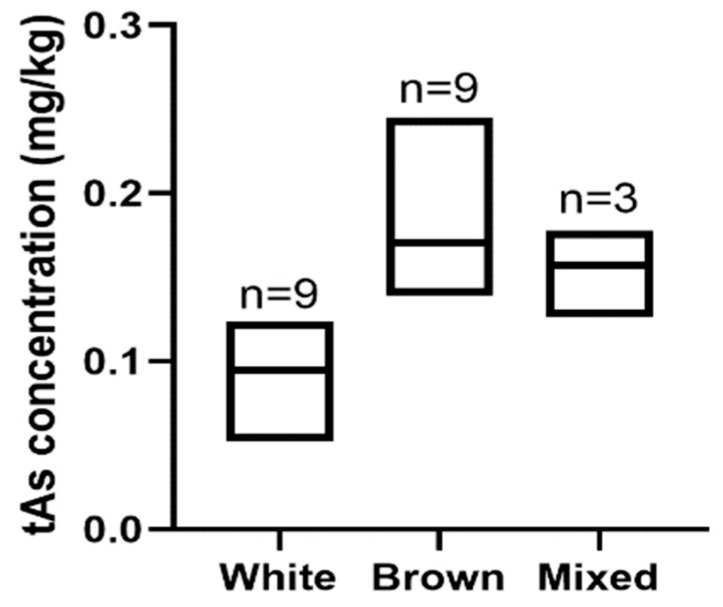
Box plot with line at mean for total arsenic (tAs) concentrations for rice crackers made from white, brown and both white and brown rice (mixed).

For rice crackers, the mean tAs concentration (0.17 mg kg^−1^) of brown rice was significantly higher than that of white rice (0.095 mg kg^−1^) (Figure 3: *p* = 0.02). The lower As concentration in white rice crackers was most likely due to the outer layer (rice bran) that tends to contain high As being removed during the polishing process [14,15]. Our finding of higher tAs in brown rice-containing foods is consistent with Rahman et al. [13] and Meharg et al. [15]. This finding is of particular concern because brown rice is generally considered a healthier choice due to its higher fibre and nutrient contents.

The remaining food groups did not have enough samples of both white and brown rice composition to undertake analysis on such relationships between tAs and rice type.

#### 3.2.4. The Relationship between tAs Concentration and Country of Origin of Products

**Figure 4:**
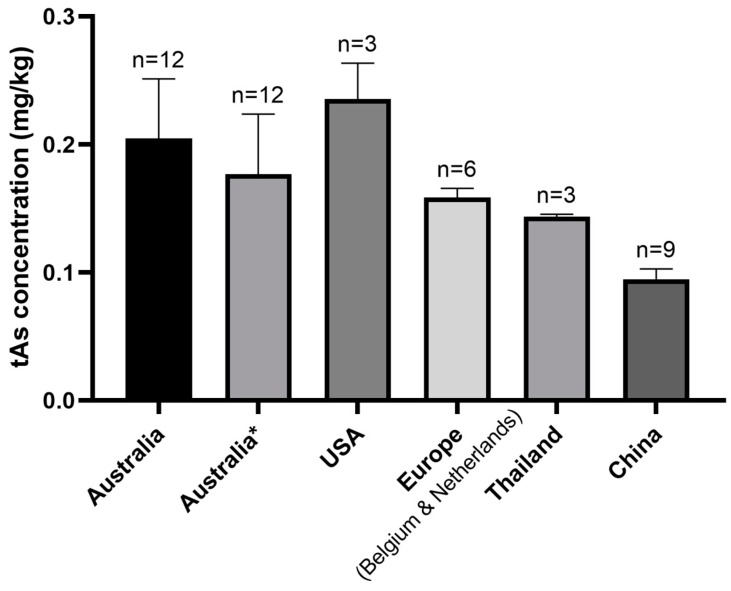
Mean and standard error of tAs concentrations of infant rice-based foods from different countries of origins of products purchased in Melbourne, Australia.

Infant rice-based foods imported from China had the lowest mean tAs concentration (0.10 mg kg^−1^) of all the countries of origins of products tested (Figure 4), probably as a result of strict national maximum level in China for As in rice (0.15 mg kg^−1^ for iAs) [36]. This was followed by Thailand and European countries (Belgium and the Netherlands). The European Union also has relatively strict maximum levels and established the first designated level for As in rice for infants and children [22]. In comparison, products imported from the United States of America (USA) had the most elevated mean tAs concentration (0.24 mg kg^−1^), possibly due to the USA being one of the countries affected by tAs contamination, particularly in groundwater in certain areas, which is used in irrigation [37]. Moreover, there are no maximum levels for As in the USA [38]; however, a recently proposed draft guidance suggests an action level of 0.1 mg kg^−1^ for iAs in rice cereal for infants [39].

Rice-based foods produced in Australia and products made from Australian and imported ingredients had mean tAs concentrations of 0.21 mg kg^−1^ and 0.18 mg kg^−1^, respectively. This is consistent with previous Australian results for rice-based foods [23]. The high tAs mean concentration in Australia probably resulted from the higher As concentrations in Australian rice than imported rice [13,24,40] and the higher Australian maximum level for tAs in rice compared to many countries (Table 1).

### 3.3. Dietary Exposure Estimation

#### Dietary Exposure Based on Australian Mean Food Consumption Rate

The tAs dietary exposure was rice pasta^a^ > rice crackers^b^ > rice cereal^c^ > rice milk powder^c^ (values with different superscript letters are significantly different at *P* < 0.05) (Table 4). The iAs dietary exposure was rice pasta^a^ > rice crackers^a^ > rice milk powder^b^ (values with different superscript letters are significantly different at *p* < 0.05) (Table 4).

For a 9-month-old infant, the tAs mean dietary exposure of all food groups was below the range of BMDL01 (0.3–8 μg kg^−1^ b. wt. day^−1^) [12] (Table 1 and Table 4). However, in terms of 90th percentile exposure, 33% of rice cracker samples and all rice pasta samples were within the range of BMDL01 of 1% extra risk (0.3–8 μg kg^−1^ b. wt. day^−1^) (Table 1 and Table 4). The iAs dietary exposure analysis showed that mean exposure for all food groups tested was below the range of BMDL01; however, for rice pasta, the 90th percentile dietary exposure of iAs was within the range of BMDL01. The results indicate that infants consuming a high number of portions of rice pasta and/or rice crackers may have increased risk of health impacts associated with excess consumption of tAs and iAs through dietary exposure. The popularity of rice-based infant snack foods in Australia and the increasing incidence of infants with wheat intolerances [41] suggest that this type of scenario is becoming increasingly likely in Australia and potentially globally. In comparison, even though infant rice milk powder had relatively high tAs and iAs concentrations (Table 2), it was unlikely to cause any detrimental effect on infants and toddlers when reconstituted with water as suggested by the manufacturer.

For 2–5 year-old children, the dietary exposure was generally 1.6-fold higher than that of a standard 9-month old infant, which could be attributed to a generally higher food consumption resulting in the higher consumption of rice products in their diet (Table 4). For tAs dietary exposure, all mean exposures were below the range of BMDL01 range, while rice pasta and crackers’ 90th percentile exposure of tAs were within the range of BMDL01 (Table 1). For iAs analysis, only the 90th percentile exposure for rice pasta was within the range of BMDL01 (Table 4). Based on the results, for 2–5 year-old children in Australia, rice pasta and crackers may have an unacceptable risk of arsenic dietary exposure when consumed in high quantities while rice milk powder and cereal are unlikely to pose any risk. Again, due to consumer preferences and increasing incidences of wheat intolerance, this is becoming an increasingly likely consumption pattern in Australian children [41].

## 4. Conclusions

Rice-based products are widely used to feed infants and children due to a number of advantages, including its bland taste, fortified iron and low allergic potential. However, the research presented here demonstrates that the concentrations of iAs and tAs in some rice-based infant foods sold in Australia are concerning for children consuming multiple serves of these products on a regular basis. In addition, the current Australian guidelines for As in food are based on adult consumption and are not protective of high-sensitivity consumers like infants and young children. This study concludes that there may be unacceptable risks due to elevated As consumption for Australian infants consuming a large amount of rice-based products, especially rice pasta and rice crackers, in their diet.

## Figures and Tables

**Table 1:** Guidelines for arsenic concentration and arsenic dietary exposure.

Year of Implementation	Name	Concentration	Jurisdiction	Reference
**As Concentration in Food (mg kg^−1^)**				
2017	Permissible limit of tAs in rice and rice cereal	1.0	Australia	[24]
2012	Proposed maximum levels for tAs in rice	0.3	FAO/WHO	[25]
2014	Maximum levels for iAs in rice	0.2	FAO/WHO	[26]
2016	Maximum levels of iAs in food for infants and young children	0.1	EU	[22]
**As Dietary Exposure (μg kg^−1^ b. wt.)**				
1967	Maximum tolerable daily intake (MTDI) for tAs	50	FAO/WHO (Withdrawn)	[27]
2010	Benchmark dose lower confidence of 0.5% (BMDL0.5)	3	FAO/WHO	[28]
2009	Benchmark dose lower confidence of 1% (BMDL01)	0.3–8.0	EU	[12]

**Table 2:** Total arsenic (tAs), inorganic arsenic (iAs) concentrations (mg kg^−1^ f.w.) and the proportion of iAs in rice-based infant foods on a fresh weight (f.w.) basis (values are mean ± standard error).

Product Category	n	Mean As Concentration		Mean iAs %
		tAs	iAs	
Rice milk powder	3	0.428 ± 0.002	0.160 ± 0	37.4 ± 0.43
Non-rice milk powder	3	<0.004	−	−
*P*-value		<0.001		
Rice pasta	3	0.186 ± 0.003	0.155 ± 0.005	84.8 ± 8.40
Non-rice pasta	3	0.006 ± 0.002	−	−
*P*-value		<0.001		
Rice cereal	12	0.134 ± 0.023	−	−
Rice crackers	21	0.132 ± 0.010	0.094 ± 0.012	74.3 ± 1.73

**Table 3:** Comparison of tAs and iAs concentrations (mg kg^−1^) in infant rice-based foods in the current study and the published literature (values are mean ± standard error, range in brackets).

Product Category	Sampling Place	n	Mean As Concentration		Reference
			tAs	iAs	
Rice milk	Australia	3	0.428 ± 0.002 ^1^/0.020 ± 0 ^2^	0.160 ± 0 ^1^/0.008 ± 0 ^2^	Present study
	Switzerland	6	0.015 (0.011–0.025) ^3^	0.009 (0.007–0.013) ^3^	[35]
Rice pasta	Australia	3	0.186 ± 0.003	0.155 ± 0.005	Present study
	Spain	4	0.192 (0.132–0.285)	0.136 (0.794–0.170)	[19]
Rice cereal	Australia	12	0.134 ± 0.023	–	Present study
	Australia	2	0.268 ± 0.006	0.073 ± 0.006	[23]
	United States	105	0.132 (0.050–0.723)	0.091 (0.023–0.283)	[16]
	United Kingdom	53	0.119 ^1^ (0.042–0.396)	0.075 (0.008–0.323)	[11]
	Switzerland	7	0.278 (0.065–0.630)	0.204 (0.046–0.331)	[35]
	Spain	9	0.309 ± 0.011	0.107 ± 0.021	[9]
Rice crackers	Australia	21	0.132 ± 0.010	0.094 ± 0.012	Present study
	United States	199	0.121 (0.009–1.931)	0.079 (0.008–0.273)	[16]
	United Kingdom	97	0.141 ^4^ (0.019–0.328)	0.111 (0.018–0.211)	[11]
	Switzerland	25	0.168 (0.047–0.361)	0.134 (0.040–0.279)	[35]

^1^ rice milk samples in powder form; ^2^ The As concentrations were recalculated based on manufacturer-recommended serving size in order to compare with liquid rice milk. ^3^ Rice milk samples in liquid form; ^4^ The tAs result from Signes-Pastor et al. [11] were based on ΣAs species and therefore, should only be used as an indication for tAs.

**Table 4:** Mean and 90th percentile dietary exposure of total arsenic and inorganic arsenic (μg kg^−^^1^ b. wt. day^−^^1^) for 9 month and 2–5 year-old children.

Food Category	n	9 Month				2–5 Years			
		tAs		iAs		tAs		iAs	
		Mean	90th Percentile	Mean	90th Percentile	Mean	90th Percentile	Mean	90th Percentile
Rice milk powder	3	0.02	0.04	0.007	0.015	0.03	0.06	0.01	0.02
Rice pasta	3	0.18	0.36	0.15	0.30	0.29	0.58	0.24	0.48
Rice cereal	12	0.03	0.06	−	−	0.05	0.10	−	−
Rice crackers	21	0.13	0.26	0.09	0.18	0.21	0.41	0.15	0.29

## Supplementary Materials

The following are available online at https://www.mdpi.com/1660-4601/17/2/415/s1, Figure S1: Relationship between total arsenic (tAs) concentration and the proportion of rice in (a) infant rice milk; (b) infant rice cereal; (c) infant rice pasta.
