# Supplementary material for: Arsenic Concentrations and Dietary Exposure in Rice-Based Infant Food in Australia
Source: Int J Environ Res Public Health. 2020 Jan 8;17(2):415. doi: 10.3390/ijerph17020415 (PMC7014030; doi:10.3390/ijerph17020415)
Supplement: Supplementary file 1 [file ijerph-17-00415-s001.pdf]

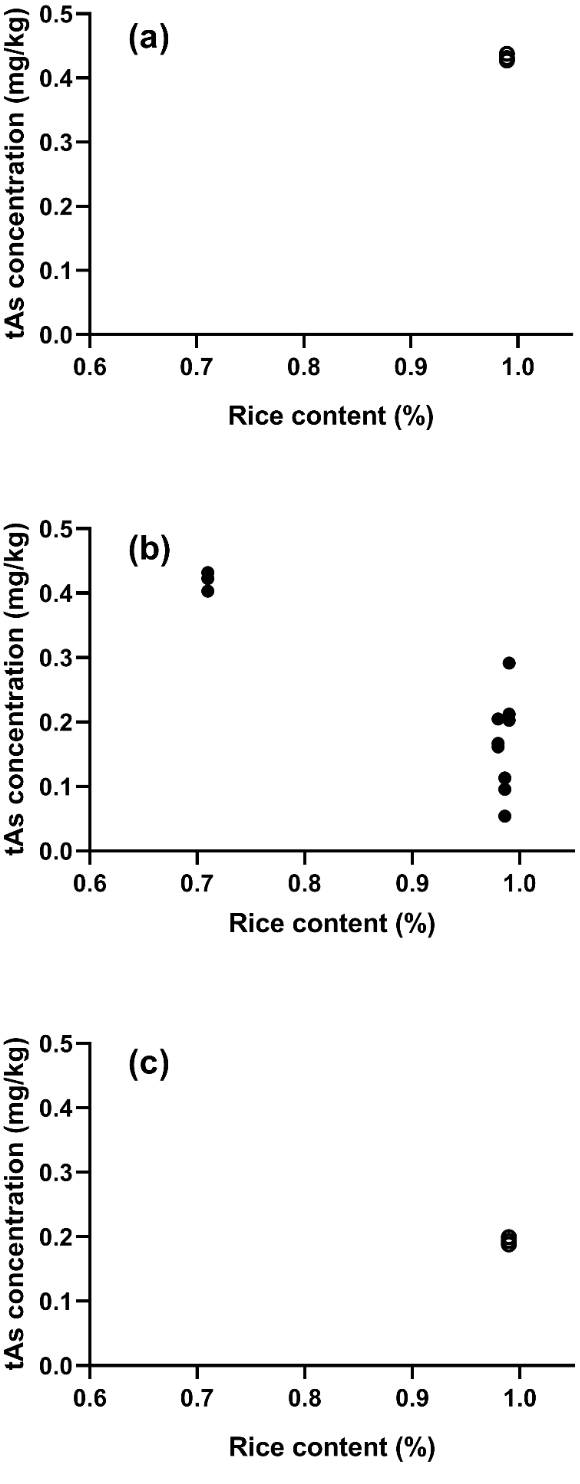

Figure S1: Relationship between total arsenic (tAs) concentration and the proportion of rice in (a) infant rice milk; (b) infant rice cereal; (c) infant rice pasta.
